# Erratum: Elshazli et al. Laser Surface Modification of TC21 (α/β) Titanium Alloy Using a Direct Energy Deposition (DED) Process. *Micromachines* 2021, *12*, 739

**DOI:** 10.3390/mi12091078

**Published:** 2021-09-07

**Authors:** Ahmed Magdi Elshazli, Ramadan N. Elshaer, Abdel Hamid Ahmed Hussein, Samar Reda Al-Sayed

**Affiliations:** 1 Department of Engineering Applications of Laser, National Institute of Laser Enhanced Sciences (NILES), Cairo University, Giza 12611, Egypt; eg.engshazly@yahoo.com; 2 Department of Mechanical Engineering, Tabbin Institute for Metallurgical Studies (TIMS), Cairo-Egypt, Helwan 11731, Egypt; ramadan_elshaer@yahoo.com; 3 Department of Metallurgy, Faculty of Engineering, Cairo University, Giza 12611, Egypt; aahussein41@yahoo.com

In the original article [1], there was a mistake in Figure 9 as published. It was repeated with Figure 14. The corrected Figure 9 appears below.

The authors apologize for any inconvenience caused and state that the scientific conclusions are unaffected. The original article has been updated.

## Figures and Tables

**Figure 9:**
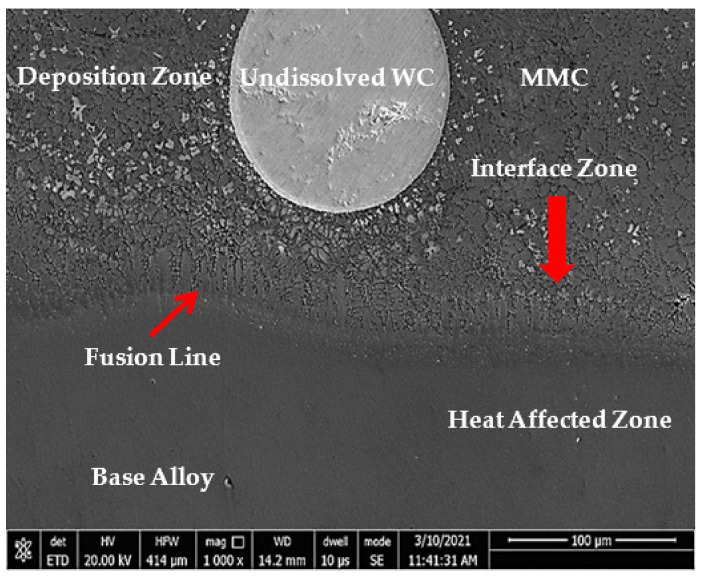
FESEM micrograph of S2 cross section of the whole deposited layer showing three different zones of deposition, interface and HAZ.
